# Breaking the state‐of‐the‐art in the chemical industry with new‐to‐Nature products *via* synthetic microbiology

**DOI:** 10.1111/1751-7915.13372

**Published:** 2019-01-31

**Authors:** Laura Martinelli, Pablo I. Nikel

**Affiliations:** ^1^ IN SRL 33100 Udine Italy; ^2^ The Novo Nordisk Foundation Center for Biosustainability Technical University of Denmark 2800 Lyngby Denmark

Scientists study the world as it is; engineers create the world that has never beenTheodore von Kármán (1881–1963)

The core tenet of synthetic biology is applying engineering principles such as standardization, modularity and rational design to accelerate the *design‐build‐test* loop aimed at reprogramming biological systems by endowing them with novel tasks (Endy, 2005). This circumstance has been broadly exploited to engineer whole‐cell catalysts able to produce a plethora of added‐value molecules (Lee *et al*., 2012; Nielsen and Keasling, 2016; de Lorenzo *et al*., 2018). Accordingly, the last few years have witnessed a steady increase in the number and type of molecules that can be accessed through rational modification of biocatalysts (Renata *et al*., 2015; Smanski *et al*., 2016; Arnold, 2018) – after all, and as recently pointed out by Prather (2019), biology is a most remarkable and versatile chemist. Yet, only a very limited number of structurally simple metabolites (e.g. the diols 1,4‐butanediol and 1,3‐propanediol) and a few natural active compounds (e.g. artemisinin) have found their way towards industrial‐scale production (Calero and Nikel, 2019). Indeed, commercially relevant bioprocesses account for a mere 3.5% of the total production volume of commodity and specialty chemicals nowadays (Campbell *et al*., 2017). Accessing new‐to‐Nature products through synthetic microbiology is not only desirable, but also an actual necessity in a rapidly changing world in which the access to natural, fossil‐based resources is becoming critically limited.

The European chemical industry can definitely benefit from the purposeful redesign of the ‘biochemical palate’ of bacteria to access products that are difficult to obtain otherwise. Bringing non‐biological chemical elements into the biochemical agenda of cell factories, e.g. halogens and silicon (O'Hagan and Deng, 2015; Kan *et al*., 2016), has the potential of *actually* revolutionizing bioproduction by multiplying the catalytic power of whole‐cell biocatalysts. The biological incorporation of fluorine (F) into organic molecules is a particularly fascinating possibility to break the state‐of‐the‐art in the chemical industry. Current technologies for addition of F into organic structures rely on chemical reactions derived from non‐renewable sources, and often require corrosive reagents (Harsanyi and Sandford, 2015) – with the corresponding negative environmental impact that they cause. The expansion of the market of fluorinated molecules in the pharmaceutical [ca. 25% of the drugs currently licensed contain some type of fluorinated structure (Wang *et al*., 2014)], agriculture and material sectors worldwide (Fig. 1) is not matched by the development of more efficient, safer and milder technologies needed for their production. The global market demand of fluorochemicals is expected to reach >5300 kiloTons by 2024, expanding at a compound annual growth rate of 4.3% during the period 2014–2024. Because of the broad applications of fluoropolymers in essentially all industrial sectors, these fluorinated compounds represent >50% of the increase in the overall demand of fluorochemicals (Fig. 1). Indeed, the global fluoropolymer market was valued at 4700 M€ in 2015, and is expected to reach >7800 M€ by 2022, registering a compound annual growth rate of 7.7% from 2016 to 2022. The Asia–Pacific axis accounted for a >45% share of the total revenue of fluorochemicals in 2015, followed by North America and then Europe. Moreover, developing countries in the Asia–Pacific region are anticipated to register steady growth rates in their individual economies owing to the increasing growth of the overall, worldwide economy. What is the role of synthetic microbiology in tackling these issues, and positioning Europe as a leader region for innovation towards a true bioeconomy based in new‐to‐Nature products?

**Figure 1:**
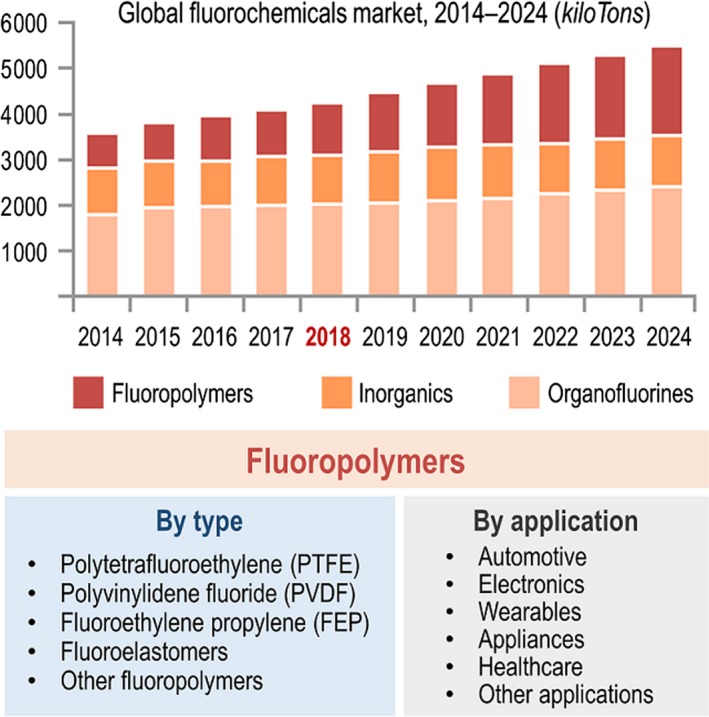
Global market of fluorochemicals and classification and applications of fluoropolymers. The current trends indicate a steady increase in the global market demand for diverse fluorochemicals and, in particular, fluoropolymers. As indicated in the inset, these molecules have countless applications in almost every sector of the industry. Thus far, no bio‐based technologies were available for the production of fluorochemicals.

As of today, there are no alternatives to traditional F chemistry available for production, and classical chemistry approaches have largely failed to provide an economically and environmentally sustainable route to fluorochemicals. Against this background, the EU project *SinFonia* (Synthetic biology‐guided engineering of *Pseudomonas putida* for biofluorination), coordinated by P.I.N., brings together 13 partners [including 5 small‐to‐medium enterprises (SMEs) and a large multinational company] with the common goal of providing a bio‐based solution for the production of fluorochemicals (Fig. 2). *P*. *putida* is the bacterial *chassis* of choice to engineer *trans*‐metabolisms for the production of this sort of molecules (Nikel and de Lorenzo, 2018), which require a complex biochemistry that few (if any) bacteria can support. The scope of *SinFonia*, a *Research and Innovation Action* embedded in the *BIOTEC‐03‐2018―Synthetic biology to expand diversity of nature's chemical production* call, will set the stage for a future economically, ecologically and societally sustainable value chain for the production of novel, bio‐based fluoropolymers from renewable substrates. In particular, the markets addressed in the domain of fluoropolymer applications are key for some of the most vital value chains for Europe competitiveness, i.e. electronics, automotive and textiles. Synthetic microbiology lays at the core technologies to be implemented in *SinFonia* to enable novel biochemistries in the platform strain KT2440.

**Figure 2:**
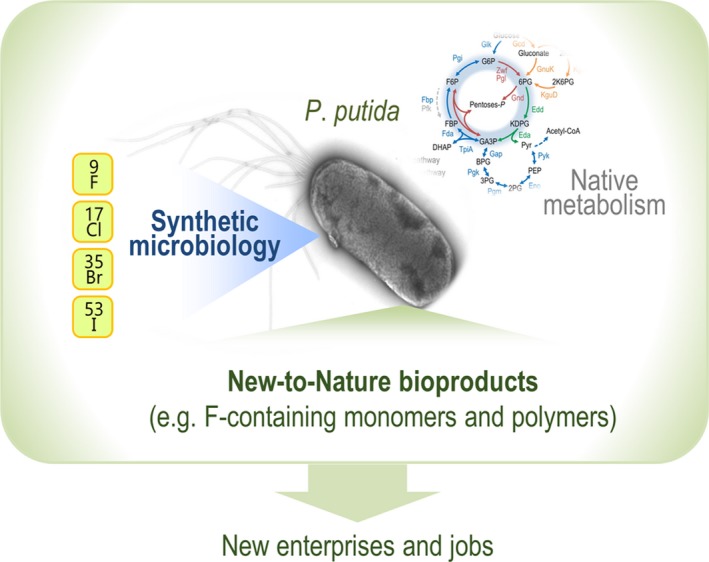
Synthetic microbiology as the enabling technology for the production of new‐to‐Nature products. The EU‐funded project *SinFonia* (Synthetic biology‐guided engineering of *Pseudomonas putida* for biofluorination) is a transnational effort aimed at developing novel biochemistries in the platform strain *P*. *putida* KT2440. Synthetic microbiology will bring non‐biological elements, such as halogens, into the core metabolism of this bacterium for the sake of whole‐cell catalysis, resulting in new‐to‐Nature products containing fluorine. The ultimate outcomes of the project include the creation of bio‐based enterprises and jobs towards a sustainable EU bioeconomy.

Meanwhile, what is the situation of biotechnology‐based companies in Europe to broaden their scope through synthetic microbiology? As recently indicated by Vilanova and Porcar (2019), bio‐based production keeps growing in number and revenues in the EU, and the market, traditionally controlled by a handful of big chemical and pharma companies, is expanding through the activities of SMEs that developed creative business models based on research and development as the pillar of competitiveness. UK and Germany form a powerful hub of biotechnology companies – in 2017, around 30% of all venture capital in Europe went to activities based in UK. Scandinavian countries are likewise strong promoters of innovation in the industrial sector. The so‐called *Medicon Valley* is a leading international life sciences cluster in Europe, spanning the Greater Copenhagen region of eastern Denmark and southern Sweden and composed by a large number of life science companies and research institutions. Life science companies in Medicon Valley employed 41 300 individuals in 2015, registering an annual increase in this trend of around 4%. Denmark leads the way in biotechnology‐oriented companies: in 2016, for instance, the Danish biotechnology sector was classified as the best in Europe, only behind United States and Singapore globally (Morrison and Lähteenmäki, 2017). Research and innovation in the Scandinavian region (and in Denmark in particular) is fuelled by >2000 M€ of tax contribution. Technology transfer is also an important part of the region‐wide initiative: the number of patent applications submitted to the European Patent Office by Danish life science companies rose 16% between 2008 and 2016. Taken together, these figures indicate that the ground for novel, biotechnology‐based (and synthetic biology‐inspired) entrepreneur endeavours in the Scandinavian region is flourishing. EU‐funded research and innovation actions, which bring together diverse academic expertise as well as technical and industrial known‐how, will be major facilitators for the much sought academia‐to‐industry transition.

The technology transfer to the industrial sector could take place in many ways, but there are mainly two models of value chain development of bio‐based processes. The first model is *absorption*, where parts or all of the value chain of synthetic microbiology are used within a sector‐specific company. This approach implies that an acquisition of facilities, human resources and knowledge is pursued by the company that is aiming to bring the new product(s) and/or process(es) to the market. This also implies either a profound organizational change (usually an extremely complex process in a company – the success of which cannot be taken for granted), or the acquisition of an entire new company (basically an SME). *Selling‐in*, in contrast, does not require a transformational change: a product is designed in a company and sold into the production step of a specific sector. The challenge here is to properly address the question ‘what is to be sold and to whom’. The two strategies can be implemented depending on the dynamics of the market targeted – and, again, a fertile ground for the development of spin‐offs and drop‐in companies is a pre‐requisite for the smooth transition of knowledge‐based technologies into actual applications in the industrial sector. The adoption of one model or the other will have a profound influence on this rapidly growing field, shaping the value chain ecosystem that will emerge in the next 20–30 years. The ecosystems resulting from the different options are substantially different; in the first case (*absorption*)*,* it will be the multinationals to lead the change, with spin‐offs and start‐ups providing the seeds of innovation without ever growing to a fully mature stage. In the second scenario (*selling‐in*), it is more likely that a more diverse ecosystem will emerge, sustaining new companies to grow to a mature stage. Currently in Europe, the pharmaceutical industry has shown preference for the *absorption* model, while the chemical industry is still waiting to see the first large batch of bio‐based commodities to be developed – and which of the options will be preferred in this case is still a matter of debate. Considering the lessons learnt from the first wave of synthetic biology‐related products and the public debate associated, it is likely that a mixed scenario will emerge, and this would be also be preferable and more sustainable in the long run.

One way or the other, some long‐sought goals for sustainable bio‐based production of chemical building blocks are expected to be attained in the near future. Synthetic microbiology will not only underpin these developments, but it will also become increasingly pervasive throughout the full spectrum of research and development of new‐to‐Nature, added‐value molecules – tackling some issues that have been almost neglected in the field, such scaling up of synthetic biology constructs and processes (de Lorenzo and Couto, 2019). Combined with the creation of jobs and enterprises, synthetic microbiology holds the promise to decisively contribute to the much‐wanted shift from a petrochemical‐based to a bio‐based society and economy in Europe. Wide public acceptance will be essential in this respect; at the same time, a diverse ecosystem supported by a dynamic community of both SMEs and large companies is more likely to sustain European competitiveness.

Considering the critical point of public acceptance of disruptive technologies and science innovation, and in the particular case of synthetic microbiology, lessons can be learnt from the past, when we saw how agricultural biotechnology had met reluctance or rejection among the public. With the new technologies brought about by synthetic biology, the question many people ask themselves is whether history will repeat itself, i.e. whether there will be public controversy when the resulting products start to be commercialized (Torgersen, 2009). The commitment of the scientific community to divulge a correct and effective communication on the matter is vital in this sense. For such innovations to boost European industry competitiveness at the current stage of development, it will be essential to pursue the debate on (i) what a synthetic (micro)biology actually is, (ii) where does its value come from, (iii) what is to be sold and to whom (i.e. future value chains) and (iv) the standardization of such products (ensuring market reproducibility of the new product). Such a case‐by‐case debate should be started already at very low TRLs (technology readiness levels) and should include a coherent policy for the protection of knowledge (Chiarotti, Portaluri and Martinelli, unpublished data).

From a broader perspective, the impact on synthetic (micro)biology on the future of jobs in Europe is an important topic that raises many interesting questions, e.g. will this new wave create new and qualified jobs?, or how existing job profiles will be affected? This kind of reasoning is strictly interlinked with both the model of adoption of synthetic biology products by the industry and the impact that such products will have on the market. Some job profiles might disappear, some might require substantial re‐learning, some others will be new, including completely different skills and background knowledge. The extent and speed of this transformation is a function of business adoption, government and education – and thus a function of human decisions (Gulbranson, 2018). The scenario we have drafted implicitly considers technology development as a social process, over which many different factors play a key role. The outcome can only be forecasted by taking into consideration an integrated and wider perspective, e.g. the *Research Responsible Innovation* (Owen *et al*., 2013). Emerging in the last decade, this approach dares to take into account the difficult dilemma between thinking and reasoning on the future trajectories that an innovation can take, how to control it, and the need to allow enough freedom of movement for an innovation to unfold. Far from trying to impose limitations, we believe that considering a perspective of inclusiveness, anticipation and responsiveness to the possible impacts of synthetic microbiology by the relevant stakeholders could effectively contribute to its success as a socially positive transformation and value creation at the same time. In fact, all the scenarios discussed in this article lead to an emerging picture in EU, where the advances in synthetic (micro)biology approaches to chemistry is paired with a likewise impactful growth in the creation of new enterprises (or new activities in established enterprises) and the creation of employment – building into an authentic circular, bio‐based economy.

## Conflict of interest

The authors have no conflict of interest to declare.
